# Optimizing the dosing regimen of linezolid in critically ill septic patients undergoing continuous hemodiafiltration using a pharmacokinetic/pharmacodynamic analysis and monte carlo simulation

**DOI:** 10.1186/2197-425X-3-S1-A395

**Published:** 2015-10-01

**Authors:** H Barrasa González, A Martín López, A Isla Ruiz, A Rodríguez Gascón, A Soraluce Olañeta, E Asín Prieto, A Canut Blasco, JA Sánchez Izquierdo, B Fernández Miret, A Vallejo De la Cueva, FJ Maynar Moliner

**Affiliations:** Osakidetza, Vitoria, Spain; University of the Basque Country, Vitoria, Spain; Doce de Octubre Hospital, Madrid, Spain

## Introduction

Pharmacokinetic (PK) of drugs in critically ill patients could vary from the general population. Patients undergoing hemodiafiltration (HDF) could present lower linezolid (LZ) concentration than expected. The pharmacokinetic/pharmacodynamic analysis (PK/ PD) is a useful tool to optimize dosing regimens of antibiotic therapy.

## Objectives

To evaluate the efficacy and safety of LZ for the treatment of infections caused by gram-positive microorganisms (GPM) in the Intensive Care Unit (ICU) patients undergoing HDF using a PK/PD analysis and Monte Carlo simulation (MCS).

## Methods

Study developed in three tertiary hospitals in patients with severe sepsis, HDF and treatment with LZ (600mg q12h). 8 each patient blood (prefilter and postfilter) and ultrafiltrate samples were taken. Concentrations of linezolid were determined by HPLC-UV. PK analysis and MCS were performed using Phoenix WinNonlin Version 6.3 (Pharsight) and Oracle Crystal Ball programs to assess the probability of successful treatment (PST) [area under the curve (AUC24)/MIC> 100 for different MICs], the probability of C_min_>2 mg/L and the risk of overexposure (RO) [C_min_> 10 mg /L and/or AUC24> 400 mg * h/L] at doses of 600mg q12 and q8h. Patients were grouped by liver and renal function considering impaired liver function (ILF) the elevation> 2 times transaminase and/or elevated bilirubin and severe renal dysfunction (SRD) the presence of CrCl < 15 ml/min. Group (G) 0: both normal, G1: ILF or SRD, G2: both. Quantitative variables were expressed as mean and standard deviation (SD), qualitative as percentages. α significance level of 0,05.

## Results

**Figure 1:**
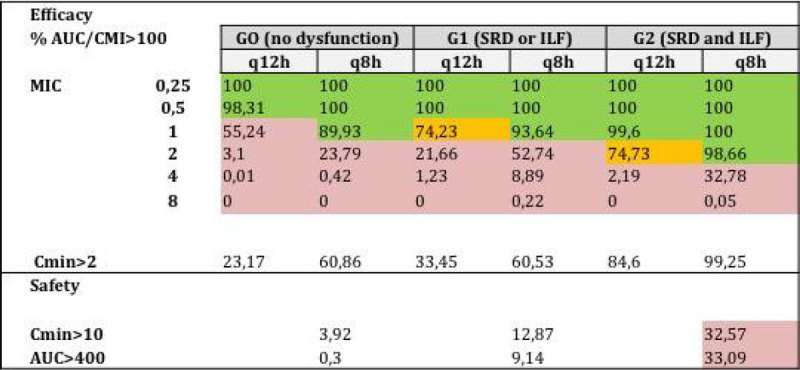
Figure 1

26 patients were included. The AUC 24 (mg * h / L) was: G0 111 (SD 39), G1 155 (SD 79) and G2 246 (SD64), the C_min_ (mg/L) was: G0 1,5 (SD 1,2), G1 2,5 (SD 1,8) and G2 4,6 (SD 2) and the clearance (Cl) (L/h) was: G0 12 (SD 4), G1 10,1 (SD 5,9) and G2 8,9 (SD 5,1). The PST was 96, 81, 38 and 0% for GPM with MICs of 0,5, 1, 2 and 4 mg/L. In the MCS, 600 mg 12qh ensures PST> 80% for MIC ≤1mg/L in the presence of some dysfunction (G1 and G2), increasing to> 90% for all groups with 600 mg q8h without RO. In the G2, the current dose assures PST> 70% for MIC of 2, increasing to >90% with 600mg q8h but with high RO (> 30%). No amount is effective for MIC ≥4 mg/L. The probability of C_min_ >2 mg/L was 23, 33 and 85% for G0, G1 and G2 respectively, increasing to 60% in G0 and G1 with 600 mg q8h (see summary table in Figure 1).

## Conclusions

In patients with RRT, 600 mg q12h guarantees PST >80% for MIC ≤1 in the presence of SRD and/or ILF. For this MIC, 600 mg q8h guarantees high PST in all patients and increases the probability of Cmin> 2. For MGP with MIC of 2, only in the presence of both dysfunctions is possible achieving the PK/PD target.

## Grant Acknowledgment

Pfizer sponsored this study.

